# A Challenging Combination: Anomalous Left Anterior Descending Coronary Artery, Myocardial Bridging, and Endothelial Dysfunction

**DOI:** 10.3389/fcvm.2020.00057

**Published:** 2020-04-15

**Authors:** Edward A. El-Am, Michel T. Corban, Amy W. Pollak, Amir Lerman, Naser M. Ammash

**Affiliations:** ^1^Department of Cardiovascular Diseases, Mayo Clinic, Rochester, MN, United States; ^2^Department of Medicine, Indiana University School of Medicine, Indianapolis, IN, United States; ^3^Department of Cardiovascular Diseases, Mayo Clinic, Jacksonville, FL, United States

**Keywords:** endothelial dysfunction, vasospasm, myocardial bridging, anomalous coronary artery, case report

## Abstract

50 years old female patient with a medical history of hypertension presented to the clinic with chest pain, palpitations, and dyspnea on exertion of 2 years duration. Extensive workup in search of the culprit etiology of her chest pain revealed a challenging combination of an anomalous left anterior descending artery with myocardial bridging and endothelial dysfunction. She was treated medically with long acting nitrates, L-arginine and calcium channel blockers, and remains asymptomatic after 12 months of follow up.

## Introduction

Ectopic origin of coronary artery (EOCA), vasospasm secondary to endothelial dysfunction (ED) and myocardial bridging (MB) can all cause angina. Congenital anomalies of coronary arteries are common in hearts with other cardiovascular malformations, such as transposition, tetralogy, or truncus arteriosus (1). They also exist in isolation in 0.5–2.2% of the population and have been implicated as a cause of sudden death especially in young athletes (1–4). Myocardial bridging, on the other hand, is more prevalent as it is noted in 40–80% on autopsy with functional MB being prevalent in up to 16% by angiography (5). It is also common to have ED within the MB leading to myocardial ischemia (5, 6). We herein report the case of a 50 years old female patient presenting with chest pain and dyspnea and found to have an unusual challenging combination of anomalous left anterior descending artery (LAD) arising from the right coronary artery (RCA) with MB and ED.

## Case Report

A 50 years old obese (BMI 32) female with a medical history significant for treated hypertension presented to the clinic with chest pain, palpitations, dyspnea on exertion and decreased functional capacity of 2 years duration. Patient reports that she has always been physically active. She denies tobacco use and any family history of sudden cardiac death.

On presentation, patient was alert, oriented and in no acute distress. She had a resting heart rate of 52 beats per minute and a blood pressure of 120/68 mmHg. On cardiac examination, her rate and rhythm were regular without audible murmurs, rubs or gallops, and no jugular venous distention. Lungs were clear to auscultation, and respirations were non-labored. The remainder of the physical examination was unremarkable.

On first visit to the clinic, electrocardiogram (ECG) showed sinus bradycardia with a heart rate of 47 beats per minute; transthoracic echocardiogram demonstrated an ejection fraction (EF) of 48% consistent with mildly diminished left ventricular systolic function without definitive regional wall abnormality. Laboratory workup was normal.

She then had a treadmill stress echocardiogram in 1 week which was negative for ischemia and showed a resting EF of 45%, an EF of 55% at peak exercise and global hypokinesis at both rest and stress. Because of her anginal chest pain and left ventricular systolic dysfunction, coronary computed tomography angiogram (CTA) was performed and the patient was started on angiotensin converting enzyme inhibitor; beta blockers were withheld because of her bradycardia.

**Figure 1:**
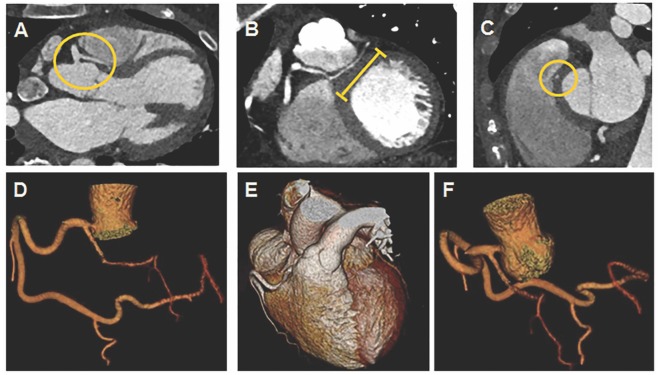
Coronary computed tomography angiogram (CTA) with 3D reconstruction. **(A)** Yellow circle denotes LAD anomalously arising from proximal RCA without a slit like orifice and coursing between aortic root and RVOT. **(B)** Yellow line denotes LAD with an intra-myocardial segment. **(C)** Yellow circle denotes LAD with an intra-myocardial segment in cross section. **(D,E)** RCA coursing into left atrioventricular groove to supply circumflex artery. **(F)** LAD arising anomalously from proximal RCA.

Coronary CTA demonstrated a very large dominant RCA coursing distally into the left atrioventricular (AV) groove to supply the circumflex (Cx) artery (Figures 1D,E); LAD arising anomalously from proximal RCA without a slit like orifice (Figures 1A,F) and coursing between the aortic root and right ventricular (RV) outflow tract (Figure 1A); LAD with an intra-myocardial segment (Figures 1B,C). Coronary CTA showed no coronary calcification or stenosis.

**Figure 2:**
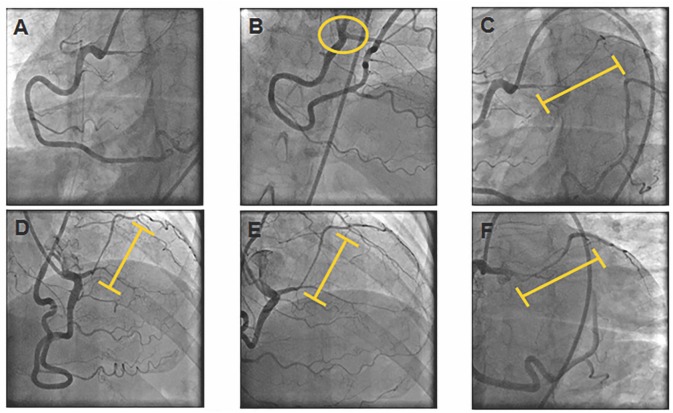
Coronary Angiogram. **(A)** RCA providing blood flow to the circumflex and obtuse marginal arteries. **(B)** Yellow circle denotes LAD arising anomalously from proximal RCA. **(C,D)** Yellow line denotes LAD myocardial bridge with spontaneous vasospasm and vasospasm of distal RCA. **(E,F)** Improvement of intramyocardial LAD segment and distal RCA calibers after intracoronary nitroglycerine.

She was started on non-dihydropyridine calcium channel blockers (CCB); however, her symptoms persisted on 1 month follow up and therefore a coronary angiogram was performed to assess extent of LAD MB and possible presence of vasospasm/ED in preparation for potential surgery to treat anomalous LAD. Coronary angiogram showed a superdominant RCA providing blood flow to the Cx and obtuse marginal arteries (Figure 2A); LAD arising from proximal RCA (Figure 2B) with a long bridged segment (intramyocardial course) with spontaneous vasospasm, along with vasospasm of distal RCA segments (Figures 2C,D). With intracoronary nitroglycerine, the intramyocardial LAD segment and distal RCA vessel calibers improved (Figures 2E,F), consistent with diagnosis of vasospasm secondary to ED.

Given her anomalous left coronary artery and the extent of LAD MB, cardiac surgery were consulted and the decision was to treat medically with long acting nitrates, L arginine and a higher dose of CCB. That decision was based on the fact that her LAD was relatively a small artery with very long segment of MB that we did not believe would be amenable for surgery. Patient was also instructed to avoid smoking and vigorous exercise. On follow-up at 1 and 12 months, patient has been overall well-managed with medical therapy, remains asymptomatic and tolerating her medications well.

Table 1 summarizes the timeline of events.

**Table 1:** Timeline of events.

At presentation	• Chest pain, palpitations, dyspnea on exertion and decreased functional capacity of 2 years duration • ECG showing sinus bradycardia • TTE demonstrating an ejection fraction of 48% with borderline to mildly diminished left ventricular function • Normal laboratory workup
1 week after presentation	• Treadmill stress echocardiogram negative for ischemia and showing a resting EF of 45%, an EF of 55% at peak exercise and global hypokinesis at both rest and stress • Started on angiotensin converting enzyme inhibitor
3 weeks after presentation	• Coronary CTA showing: ∘ Very large RCA coursing distally into the left AV groove to supply the circumflex ∘ LAD arising anomalously from proximal RCA without a slit like orifice ∘ LAD intra-arterial between aortic root and RVOT ∘ LAD with an intra-myocardial segment ∘ No coronary calcification or stenosis • Started on CCB
2 months after presentation	• Persistent symptoms despite being on CCB • Coronary angiogram showing: ∘ Superdominant RCA providing blood flow to the circumflex and obtuse marginal arteries ∘ LAD arising from proximal RCA has a long bridged segment with vasospasm, along with spasm of distal RCA segments ∘ Intracoronary nitroglycerin increased intramyocardial vessel and distal RCA caliber suggesting vasospasm/ED • Started on long acting nitrates, L-arginine, and higher dose of CCB
3 months and 1 year after presentation	• Asymptomatic

*ECG, electrocardiogram; TTE, transthoracic echocardiogram; EF, ejection fraction; CTA, computed tomography angiogram; RCA, right coronary artery; AV, atrioventricular; LAD, left anterior descending; RVOT, right ventricular outflow tract; CCB, calcium channel blocker; ED, endothelial dysfunction*.

## Discussion

Anomalous LAD arising from RCA and having an intra-arterial course has been associated with sudden death and often requires surgical intervention (7). This case illustrates that although CT coronary angiography can delineate the current anatomy very well in patients with ectopic coronary artery origin, at times as illustrated in this case, coronary angiography provides an added value to the evaluation especially in presence of MB. We usually do vasoreactivity testing using acetylcholine to evaluate for epicardial and microvascular dysfunction; however, in our case given the amount of spontaneous vasospasm within the bridge, and the associated high prevalence of endothelial dysfunction with MB, we didn't pursue a vasoreactivity test as we felt it would be unsafe to further induce vasospasm. In addition, this case demonstrates the clinically challenging association of EOCA with MB and ED in a symptomatic patient with angina who improved with medical therapy rather than surgical intervention. ED and/or vasospasm can occur within MB and can be associated with EOCA. They can be successfully treated with nitrates, L-arginine (8) and CCB.

## Conclusion

Symptomatic patients with EOCA should routinely be considered for invasive assessment of coronary vasoreactivity to identify culprit etiology before considering any surgical intervention. ED and/or vasospasm within MB could be associated with EOCA and be treated medically. First line therapies for ED are nitrates, L-arginine and CCB, while first line therapies for symptomatic MB are CCB and BB.

## Data Availability Statement

The datasets generated for this study are available on request to the corresponding author.

## Ethics Statement

This case report was exempted from any ethics committee verification due to its retrospective nature. Consent was obtained from the patient to use the images and case presentation for publication. Publication of this material was approved by the institutional review board under exempt category.

## Author Contributions

All authors contributed to the writing and revision of the manuscript.

### Conflict of Interest

The authors declare that the research was conducted in the absence of any commercial or financial relationships that could be construed as a potential conflict of interest.
